# Supplementary material for: Predictors of severity and development of critical illness of Egyptian COVID-19 patients: A multicenter study
Source: PLoS One. 2021 Sep 23;16(9):e0256203. doi: 10.1371/journal.pone.0256203 (PMC8459940; doi:10.1371/journal.pone.0256203)
Supplement: S1 Table — (DOCX) [file pone.0256203.s001.docx]

**Supplementary Table 1: The difference in clinical and Laboratory characteristics of the included patients according to gender.**

| Parameters | Female | Male | P-value |
| --- | --- | --- | --- |
| Critical Illness | 202 (47.75%) | 221 (52.25%) | P=0.655 |
| Age > 60 years | 560 (50.68%) | 545 (49.32%) | P=0.096 |
| Smoking | 325 (96.44%) | 12 (3.56%) | **P<0.001** |
| Contact History | 238 (44.82%) | 293 (55.18%) | **P=0.043** |
| Asymptomatic | 141 (56.40%) | 109 (43.60%) | **P=0.011** |
| Fever | 926 (47.93%) | 402 (50.76%) | P=0.180 |
| Cough | 735 (45.77%) | 871 (54.23%) | **P<0.001** |
| Dyspnea | 910 (48.15%) | 980 (51.85%) | P=0.343 |
| Sore throat | 367 (46.05%) | 430 (53.95%) | P=0.069 |
| Hemoptysis | 0 (0.0%) | 10 (100%) | **P=0.002** |
| Headache | 3 (42.86%) | 4 (57.14%) | P=0.755 |
| Fatigue | 318 (46.63%) | 364 (53.37%) | P=0.20 |
| Anorexia | 31 (34.07%) | 60 (65.93%) | **P=0.004** |
| Diarrhea | 64 (37.21%) | 108 (62.79%) | **P=0.002** |
| Nausea | 58 (36.48%) | 101 (63.52%) | **P=0.001** |
| Vomiting | 65 (38.69%) | 103 (61.31%) | **P=0.007** |
| Abdominal pain | 33 (42.31%) | 45 (57.69%) | P=0.248 |
| Arthralgia | 88 (42.51%) | 119 (57.49%) | P=0.062 |
| Myalgia | 128 (48.30%) | 137 (51.70%) | P=0.877 |
| Loss of taste | 63 (49.61%) | 64 (50.39%) | P=0.844 |
| Loss of smell | 30 (33.33%) | 60 (66.67%) | **P=0.003** |
| Number of symptoms (≥7) | 1,323 (49.04%) | 1,375 (50.96%) | **P=0.002** |
| SBP | 120.16±14.96 | 121.2±14.57 | 0.107 |
| DBP | 77.21±10.09 | 78.09±10.66 | 0.215 |
| Pulse | 89.84±13.88 | 90.55±14.93 | 0.220 |
| Temperature | 37.60±0.90 | 37.67±0.81 | 0.092 |
| Respiratory rate | 23.94±11.33 | 23.20±7.49 | 0.104 |
| Oxygen Saturation | 90.53±6.96 | 89.94±7.68 | **0.046** |
| FiO2 | 29.22± 32.11 | 20.43± 28.19 | 0.109 |
| Hemoglobin (gm/dl) | 12.55±10.00 | 12.61±4.13 | 0.883 |
| Platelet count (*1000/cmm) | 262.74±123.19 | 246.9±114.73 | **0.016** |
| TLC (*1000/cmm) | 11.93±21.62 | 10.88±21.79 | 0.423 |
| INR | 0.70±0.72 | 0.65±0.74 | 0.225 |
| PTT | 38.89±27.43 | 39.63±37.98 | 0.842 |
| Serum creatinine (mg/dl) | 1.87±3.18 | 1.72±2.49 | 0.432 |
| Serum sodium (mEq/L) | 136.47±8.57 | 137.02±7.75 | 0.373 |
| Serum potassium (mEq/L) | 2.30±3.22 | 2.75±9.51 | 0.264 |
| PH | 7.39±0.24 | 7.30±0.48 | **0.009** |
| PaCO2 mmHg | 33.87±7.78 | 35.66±13.20 | 0.052 |
| HCO3 mEq/L | 24.86±21.34 | 24.20±18.27 | 0.704 |
| PaO2 mmHg | 62.41±26.72 | 60.43±25.39 | 0.395 |
| Total bilirubin (mg/dl) | 0.35±0.60 | 0.34±0.61 | 0.826 |
| Direct bilirubin (mg/dl) | 0.16±0.34 | 0.15±0.39 | 0.636 |
| Serum albumin (g/dl) | 3.73±0.73 | 3.67±0.70 | 0.373 |
| ALT (U/L) | 43.17±38.17 | 56.05±53.13 | **<0.001** |
| AST (U/L) | 54.61±68.66 | 66.08±69.90 | **0.045** |
| CRP (mg/L) | 59.26±89.25 | 62.18±76.18 | 0.739 |
| ESR (first hour) | 133.44±66.00 | 132.09±68.92 | 0.870 |
| Serum ferritin (ng/ml) | 662.36±457.10 | 865.93±577.51 | **0.002** |
| D-dimer (microgm/ml) | 1029.48±709.11 | 1371.01±946.66 | **0.002** |
| Fibrinogen (mg/dl) *100 if presented in g/L | 69.5±69.0 | 94.64±79.49 | **0.013** |
